# Evaluation of AI‐based segmentation and synthetic CT generation for MRI‐only prostate radiotherapy planning

**DOI:** 10.1002/acm2.70765

**Published:** 2026-08-25

**Authors:** Amanda Östensson, Joakim Jonsson

**Affiliations:** ^1^ Department of Diagnostics and Intervention Umeå University Umeå Sweden

**Keywords:** AI‐segmentation, Synthetic CT, MRI‐only workflow, Prostate radiotherapy

## Abstract

**Background:**

There is an increasing use of magnetic resonance imaging (MRI) for segmentation of target volumes and organs at risk due to superior soft tissue contrast compared to computed tomography (CT). Conventional workflows require CT‐MRI registration, introducing geometric uncertainty that can propagate to dose delivery. MRI‐only radiotherapy, based on AI‐generated synthetic CT (sCT) and segmentations from T2‐weighted images, offers a single‐modality approach, potentially eliminating registration errors.

**Purpose:**

Our study aims to evaluate whether an MRI‐only workflow, using commercial AI models for segmentation and sCT generation (MVision), demonstrates performance comparable to expert‐defined segmentations and reference CT‐based calculations within the studied dataset.

**Methods:**

We analyzed five segmentations on 19 male pelvis patients from the Gold Atlas dataset, providing deformably co‐registered CT and MRI, sCTs, with multi‐observer and consensus segmentations. We investigated segmentation accuracy, image similarity, and evaluated the dosimetric impact by comparing AI‐ to manual segmentations and CTs to sCTs. Wilcoxon signed‐rank tests were used for statistical significance and leave‐one‐out kappa determined segmentation outliers.

**Results:**

Results show that AI‐segmentations achieved accuracy comparable to interobserver variability across all organs. Median Dice coefficients exceeded 0.9 for the prostate and bladder, and distance metrics closely matched the manual results. The leave‐one‐out kappa showed that AI‐segmentations were indistinguishable from manual observers for all organs. sCTs showed high similarity to the reference CTs, with a median mean absolute error of 34.0 HU and median structural similarity index of 0.93. Dose recalculations showed excellent agreement; median 3D‐gamma pass rate was 99.8% (2%/2 mm), and mean absolute dose difference was 0.036 Gy. All evaluated clinical dose constraints remained fulfilled after recalculation on the sCT.

**Conclusions:**

Our results confirm that an AI‐segmentation and MRI‐sCT workflow provides geometric, image, and dose accuracy comparable to manual‐ and CT‐based references, supporting their integration into clinical workflows for pelvic radiotherapy.

## 1 INTRODUCTION

In radiotherapy (RT), there is an increasing use of magnetic resonance imaging (MRI) when creating segmentations of target volumes and organs at risk (OAR). This is due to the superior soft tissue contrast in MRI images compared to computed tomography (CT). A conventional workflow requires CT images for electron density information and MRI images for anatomical definition, which in turn requires an image registration step between the modalities. Co‐registration between CT and MRI introduces spatial uncertainties that may propagate into dose delivery.^1^, ^2^ The rationale for MRI‐only treatment planning has been motivated by the potential to eliminate CT from the workflow, thereby removing registration errors,^2^, ^3^ reducing the number of imaging sessions, improving patient comfort, reducing resource utilization,^3^, ^4^ and reducing additional ionizing dose to patients.^5^ MRI‐only workflows also enable direct application of MRI‐linac systems and adaptive strategies that exploit the superior soft tissue contrast.^6^, ^7^

Studies have shown the clinical feasibility of MRI‐only planning, especially for prostate and pelvic RT^2,^.^4^, ^8^ The reported dose differences between MRI‐only and CT‐based plans are typically within 1%–2%, where geometric uncertainties often are caused by residual distortions in the MRI and contour variability.^1^, ^9^ Therefore, methods for sCT generation have become central to MRI‐only workflows, where MRI intensities are converted into electron density equivalent images for dose calculation.^3^, ^10^, ^11^ Early atlas‐ and patch‐based approaches^12^ are being replaced by deep learning models with improved segmentation accuracy and robustness across anatomy and scanner types.^13^, ^14^, ^15^

In parallel to sCT generation, auto‐segmentation based on AI has become a key component of modern RT workflows.^16^, ^17^ Current studies indicate that deep learning segmentation networks achieve accuracy comparable to interobserver variability for several anatomical sites, with a reduction in manual workload and contouring time.^17^, ^18^ Commercial tools such as Contour+ 1.3.1 and Image+ 1.0.1 (both CE‐marked components within Workspace+ 1.0.1, MVision AI, Helsinki, Finland) provide AI‐based functions for RT planning. Contour+ delivers automatic segmentations trained on large standardized multi‐institutional datasets. Image+ Pelvis generates sCT images from T2‐weighted MRI, while the corresponding Image+ Brain model uses T1‐weighted MRI. Other commercial systems provide related solutions, including MRI‐based sCT from Siemens Healthineers and AI‐based auto‐contouring from RaySearch, among others. Our study evaluates the MVision Contour+ and Image+ models only.

Robust evaluation of such AI‐models requires standardized and clearly characterized datasets that include both imaging and expert‐defined segmentations. The Gold Atlas dataset, developed by the Swedish Gentle Radiotherapy project,^19^ provides deformably co‐registered CT and MRI image pairs, multi‐observer segmentations, and STAPLE^20^ consensus segmentations for male pelvis patients. The dataset enables direct comparison of AI and manual segmentations against a common expert‐derived reference, supporting reproducible benchmarking of segmentation and sCT accuracy. The inclusion of deformably registered CT further allows voxel‐wise comparison of dose calculations between MRI and CT, minimizing anatomical discrepancies unrelated to Hounsfield unit (HU) mapping. The dataset reports high interobserver agreement (e.g., Dice ≈ 0.90 for prostate and ≈ 0.95 for bladder), supporting its use as a reference standard.

Our study evaluates an MRI‐only RT workflow, where both AI segmentations and sCT generation were derived from T2‐weighted (T2w) MRI. Segmentations were performed using Contour+ 1.3.1 and image synthesis with Image+ 1.0.1 Pelvis, both within the Workspace+ 1.0.1 platform in MVision. Our aim is to determine whether the AI‐generated segmentations and sCTs achieve segmentation and dosimetric accuracy comparable to expert‐defined segmentations and reference CTs. This would further support the feasibility of using guidance by AI when planning for prostate and pelvic RT.

## 2 METHODS

### 2.1 Dataset

All analyses were performed on the Gold Atlas dataset,^19^ consisting of 19 male pelvis patients imaged with both CT and MRI (T1w and T2w). Patients were scanned in RT treatment position on a flat tabletop according to clinical routines, as described in the original dataset publication. The image data were acquired from three different Swedish RT departments using multiple scanner manufacturers (GE Discovery 750w, Siemens, and GE Signa PET/MR for MRI; Siemens Somatom Definition AS+, Toshiba Aquilion, and Siemens Emotion 6 for CT) with T2w sequences acquired using FRFSE (Fast Recovery Fast Spin‐Echo) or TSE (Turbo Spin‐Echo) protocols at 2.5 mm slice thickness. Detailed acquisition parameters, including scanner manufacturers, sequence types, and imaging settings, are reported in Table 1 in the original Gold Atlas publication.^19^ Five expert observers (four radiation oncologists and one radiologist) independently delineated key pelvic organs on the T2w MRI images, including the bladder, rectum, prostate, seminal vesicles, and penile bulb. Consensus reference segmentations were generated using the STAPLE (Simultaneous Truth and Performance Level Estimation) algorithm.^20^ The dataset also provides deformable registration between CT and MRI geometries to enable voxel‐wise image comparisons.

**TABLE 1:** Significant results of the Wilcoxon signed‐rank test comparing AI and manual segmentations to STAPLE consensus.

Organ	Metric	∆	*R*	*p*‐value	Sig.
Bladder	ASSD (mm)	+0.440	0.895	9.50 × 10^−5^	_***_
Bladder	Dice	−0.029	−0.745	1.17 × 10^−3^	^**^
Bladder	Precision	−0.024	−0.850	2.10 × 10^−4^	_***_
Bladder	Surface dice	−0.045	−0.574	1.24 × 10^−2^	^*^
Penile bulb	Volume difference (%)	+15.33	0.595	9.45 × 10^−3^	^**^
Penile bulb	Precision	−0.087	−0.910	7.25 × 10^−5^	_***_
Prostate	Volume difference (%)	−5.186	−0.553	1.60 × 10^−2^	^*^
Prostate	Centroid distance (mm)	+0.470	0.532	2.04 × 10^−2^	^*^
Prostate	HD95 (mm)	−0.587	−0.491	3.23 × 10^−2^	^*^
Prostate	Recall	−0.023	−0.563	1.41 × 10^−2^	^*^
Seminal vesicles	Volume difference (%)	−13.83	−0.461	4.46 × 10^−2^	^*^
Seminal vesicles	Recall	−0.072	−0.511	2.58 × 10^−2^	^*^

*Note*: Significant results of the Wilcoxon signed‐rank test that compare the AI‐based (MVision) versus mean manual (Users) segmentations, evaluated against the same STAPLE consensus segmentation. Median difference, ∆ = MVision‐UsersMean, is reported per organ, across patients. The effect size, *r* = *z*/√*n*, is shown with sign that represent the direction consistent with ∆; larger |*r*| indicates a more systematic shift across the cohort. Asterisks denote the significance level where,

^*^ = *p* < 0.05

^**^ = *p* < 0.01

^***^ = *p* < 0.001.

### 2.2 Segmentation accuracy

We assessed the segmentation accuracy by comparing AI‐generated contours (MVision) and manual delineations from five observers, against the same STAPLE consensus segmentations. This ensured that all comparisons were performed relative to a common, expert‐derived standard. For each volume, the geometric and volumetric agreement was quantified using the Dice similarity coefficient, average symmetric surface distance (ASSD), 95th percentile Hausdorff distance (HD95), surface Dice at 2 mm, centroid distance, volumetric difference, precision, and recall. Precision and recall were computed voxel‐wise, where precision represents the fraction of predicted segmentation voxels overlapping the reference segmentation, and recall represents the fraction of reference voxels captured by the segmentation.

We computed the distance‐based metrics (ASSD, HD95, and Surface Dice at 2 mm) by using a 2.5D surface‐based approach. For each segmentation, source points were defined as the in‐slice contour pixels on each axial slice, obtained slice‐wise in 2D. Distances were then measured in full 3D as the shortest Euclidean distance from those contour points to the other segmentation's 3D surface, accounting for voxel spacing. This was performed symmetrically in both directions. ASSD was calculated as the mean of the two directional mean distances, HD95 as the maximum of the two directional 95th percentiles, and Surface Dice as the fraction of contour points within 2 mm in both directions. This approach preserves the slice‐wise nature of manual contouring while evaluating distances in real 3D space.

### 2.3 Image similarity

The sCTs were generated using the Image+ 1.0.1 Pelvis model (MVision), a deep learning‐based method that converts T2‐weighted MRI into synthetic CT (sCT) images. We evaluated the sCT image accuracy relative to the reference CT by using the mean absolute error (MAE), root mean square error (RMSE), peak signal‐to‐noise ratio (PSNR), structural similarity index (SSIM), and normalized cross‐correlation (NCC). These metrics were computed voxel‐wise within the body contour for all axial slices and summarized per patient by their mean, median, and variability. To remove edge effects from distortion correction and peripheral field of view in the MRI, we excluded the top and bottom five slices from the image. To account for residual differences in body contour after deformable registration, we also eroded the body contour by five pixels.

### 2.4 Dosimetric evaluation

To determine the dosimetric impact, we evaluated the treatment plans on both the reference CT and the sCT. This was done by first creating a dual‐arc volumetric modulated arc therapy (VMAT) treatment plan on the reference CT for each patient in RayStation 2024B (RaySearch Laboratories, Stockholm, Sweden), using the Collapsed Cone algorithm. The prescription to the prostate was 42.7 Gy delivered in seven fractions. This is consistent with the Swedish RT treatment protocol for intermediate risk prostate cancer, using ultra‐hypofractionation based on the HYPO‐RT‐PC randomized, non‐inferiority, phase 3 trial published by Widmark et al.^21^ Treatment planning was performed using the STAPLE consensus structures from the Gold Atlas dataset. We generated the PTV by applying a uniform 5 mm expansion to the STAPLE prostate contour. The optimization criteria were standardized across all patients, fulfilling all relevant clinical goals. Plan geometry, monitor units, and beam parameters from the reference CT were transferred and recalculated on the sCT. The same STAPLE‐based structure set was used for DVH evaluation on both the reference CT and sCT, ensuring that the dosimetric comparison reflected differences between CT‐ and sCT‐based dose calculation rather than differences in contouring. Both the reference CT and sCT were assigned to the RayStation Generic CT HU‐to‐density calibration curve.

We evaluated dose agreement between CTs and sCTs with a combination of spatial, volumetric, and voxel‐wise metrics, capturing both global and local differences. During the voxel‐wise dose comparison and gamma analysis, we eroded the body contour by five pixels to limit the influence of residual differences in patient outline after deformable registration. We performed a 3D gamma analysis within the eroded body contour by using global normalization to the prescribed dose, with 1%/1 mm and 2%/2 mm criteria. We computed the voxel‐wise mean absolute dose difference (MADD), Dice coefficients for the 95% and 50% isodose volumes, and a continuous dose similarity index (DSI) calculated as

$$DSI=\frac{\left|\;CT_{\mathrm{Dose}}\;-\;s\;CT_{\mathrm{Dose}}\;\right|}{CT_{\mathrm{Dose}}+\;\mathrm{sC}T_{\mathrm{Dose}}}$$

Lastly, we looked at multiple parameters from the dose‐volume histograms (DVH) for target volumes and OARs, which were D_98%_, D_50%_, D_2%_, D_mean_, D_max_, D_2cc_, D_0.5cc_, and V_30 Gy_, V_36 Gy_, and V_40Gy_. DVH evaluation was performed using the same STAPLE‐based structure set on the reference CT and sCT. The evaluated volumes were bladder, external, right and left femoral heads, PTV, penile bulb, prostate, rectum, and seminal vesicles. All DVH endpoints are reported as the difference ∆ = CT—sCT (mean ± SD).

### 2.5 Analysis

We did calculations and visualizations of the data by using Hero (Hero Imaging AB, Umeå, Sweden) and Python (version 3.10), using NumPy, SciPy, and pandas.

We compared the performance of the segmentations done by AI with the mean of the five expert observers by using a paired non‐parametric Wilcoxon signed‐rank test, with *p < 0.05* considered statistically significant. Each segmentation was evaluated relative to the same STAPLE consensus. To complement the significance test, the effect size, *r = z/√n*, where *z* is the test's standardized statistic and *n* is the number of paired observations, was evaluated. The difference, ∆ = (MVision—UsersMean), effect size, and *p*‐value with a significant level are reported.

To identify which segmentation differed the most, we performed a leave‐one‐out kappa test based on Fleiss’ Kappa. We recalculated the kappa iteratively, excluding one observer segmentation at a time. An observer was considered as an outlier if their exclusion resulted in an increased kappa value.

## 3 RESULTS

### 3.1 Segmentation accuracy

**FIGURE 1:**
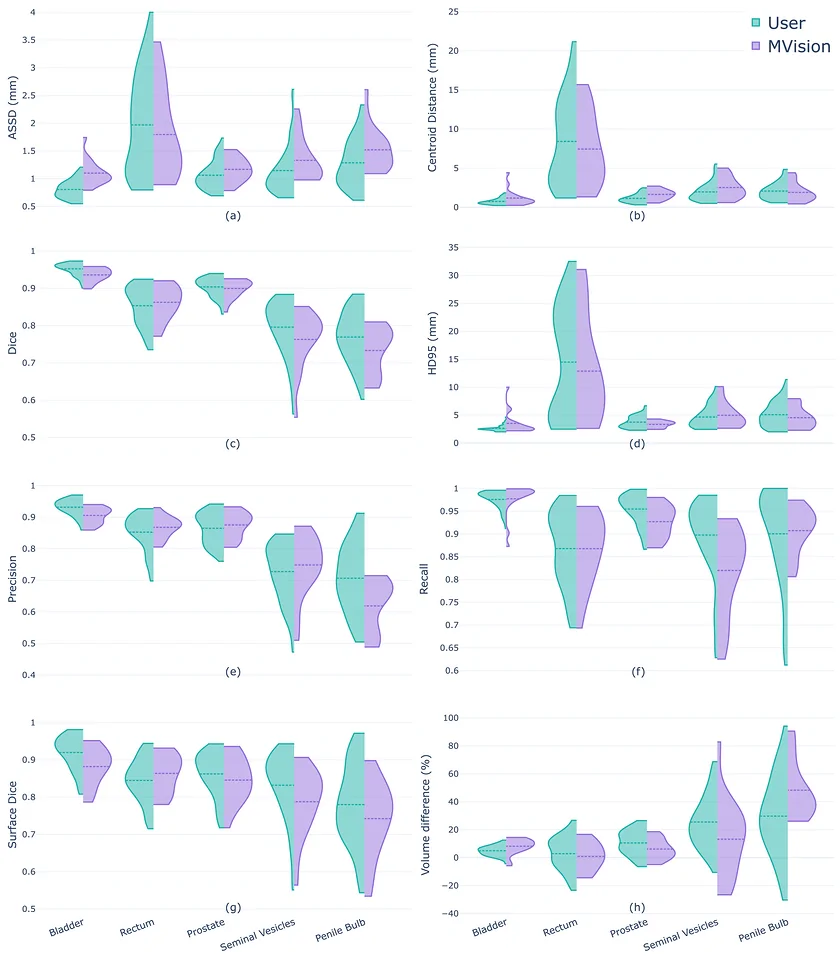
Split‐violin plot of segmentation metrics. Split‐violin plots showing the distribution of segmentation performance metrics across all patients, stratified by organ. Manual segmentations (Users) are shown in green to the left and the AI‐ segmentations (MVision) in purple to the right. The specific metrics in each subfigure are (a) ASSD, (b) Centroid Distance, (c) Dice, (d) HD95, (e) Precision, (f) Recall, (g) Surface Dice, (h) Volume difference. Each violin pools data across all patients for each given organ. Outliers were clipped at the 5th and 95th percentiles for visual clarity.

The AI‐segmentations (MVision) achieved an overall performance comparable to, or within, the range of interobserver variability across all evaluated organs (Figure 1). The mean Dice values were 0.92 for bladder, 0.90 for prostate, 0.86 for rectum, 0.76 for seminal vesicles, and 0.72 for penile bulb. The distance‐based metrics (ASSD and HD95) for the AI‐segmentations showed similar distributions as the manual segmentations. Detailed results of the segmentation metrics are found in Table S1.

Out of the 40 evaluations (five segmentations and eight metrics) used for the Wilcoxon signed‐rank test, there were 12 that had a significant result (Table 1). The significant results show small absolute median differences coupled with a medium to large |*r*| (medium > 0.5 and large > 0.8, according to Cohen's d). This is consistent with a subtle, but systematic shift across patients rather than large, isolated outliers.

**FIGURE 2:**
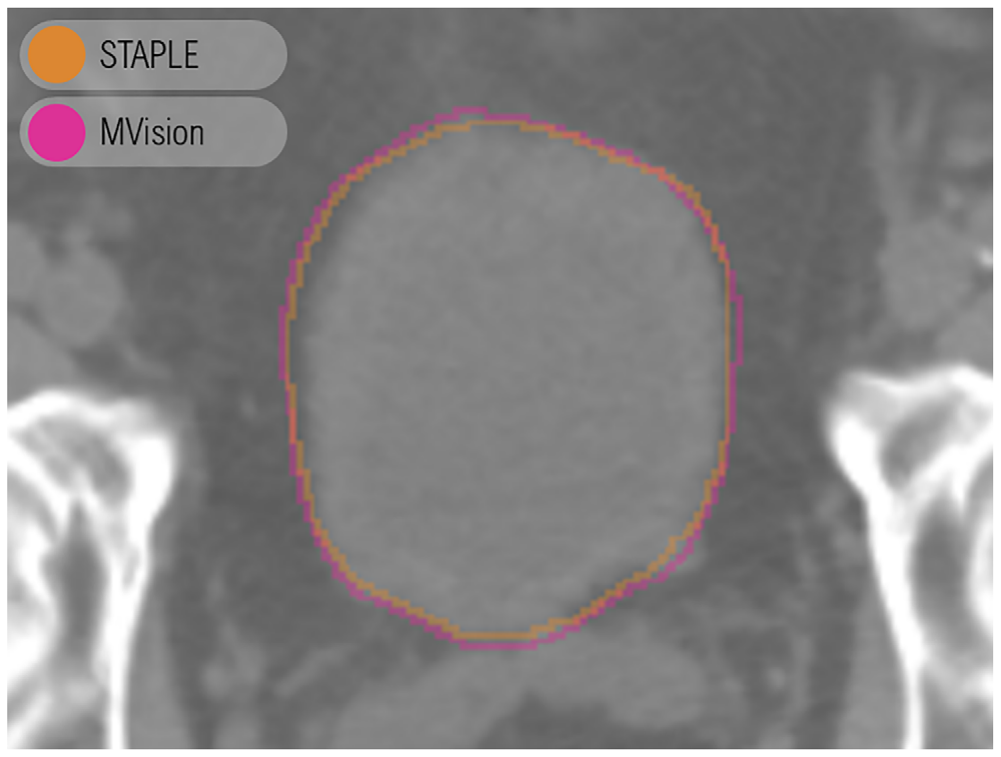
Segmentation comparison. Figure showing the segmentations of one of the patients' bladder, with the STAPLE consensus segmentation in orange and the AI‐segmentation (MVision) in pink.

A visual inspection of the split‐violin plots in Figure 1 confirms that the distribution of the AI‐segmentations largely overlapped with interobserver variability, which indicates a clinically acceptable performance across all evaluated pelvic organs. The key finding from the segmentation analysis was the leave‐one‐out kappa test (Table 2), showing that the AI‐segmentations were not distinguishable from the five manual segmentations. A visualization of the similarities is shown in Figure 2, containing the STAPLE consensus and AI‐segmentations for the bladder in one patient.

**TABLE 2:** Leave‐one‐out Kappa test to determine which observer segmentations were an outlier, stratified by organ.

Observer	Bladder	Penile bulb	Prostate	Rectum	Seminal vesicles
**MVision**	2 (10.5%)	3 (15.8%)	2 (10.5%)	0 (0.0%)	2 (10.5%)
**User1**	1 (5.3%)	3 (15.8%)	**7 (36.8%)**	1 (5.3%)	2 (10.5%)
**User2**	0 (0.0%)	1 (5.3%)	2 (10.5%)	0 (0.0%)	2 (10.5%)
**User3**	**12 (63.2%)**	**6 (31.6%)**	2 (10.5%)	4 (21.1%)	3 (15.8%)
**User4**	1 (5.3%)	**6 (31.6%)**	3 (15.8%)	**13 (68.4%)**	3 (15.8%)
**User5**	3 (15.8%)	0 (0.0%)	3 (15.8%)	1 (5.3%)	**7 (36.8%)**

*Note*: Results of the leave‐one‐out Kappa test that determine which observer segmentations were an outlier, stratified by organ. The bold value in each column represents the segmentation with highest deviation.

### 3.2 Image similarity

**FIGURE 3:**
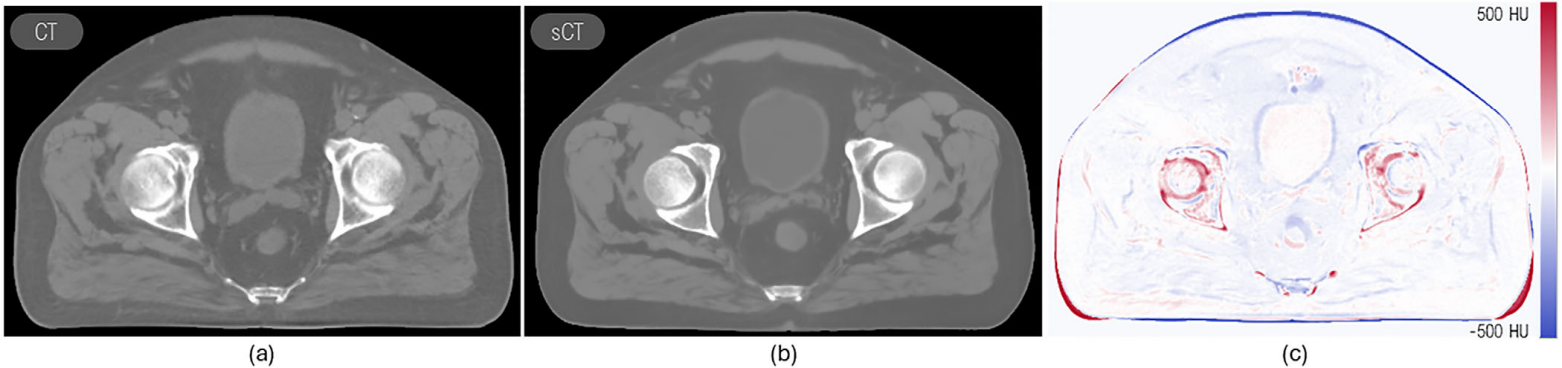
Image comparison. Figure showing in a slide from the CT in (a) and sCT in (b). The difference between CT and sCT is seen in (c).

The sCTs show a high similarity to the reference CTs across all patients. Structural metrics were consistently strong, with median SSIM = 0.93 and PSNR = 29.8 dB, while the voxel‐wise intensity errors remained moderate, with a pooled median MAE = 34.0 HU. The CT, sCT, and difference between them are visualized in Figure 3. The largest local differences were seen near the external body contour and in high‐HU bony regions, while the soft tissue differences were small. Detailed results of the image similarity metrics are found in Table 3.

**TABLE 3:** Summary of image similarity metrics comparing the sCTs to the reference CTs across all patients.

Metric	Mean	Median	SD	Min	Max
MAE [HU]	37.35	34.03	3.53	18.25	89.37
NCC	0.86	0.89	0.06	0	0.99
PSNR [dB]	29.56	29.83	0.62	20.04	40.47
RMSE [HU]	83.6	68.47	33.27	34.97	461.16
SSIM	0.92	0.93	0.01	0.65	0.99

*Note*: Summary of the image similarity metrics comparing the sCTs to reference CTs across all patients. The values represent slice‐weighted pooled estimates for the entire dataset. Higher PSNR, SSIM, and NCC indicate a greater similarity between the sCT and the reference CT, while lower MAE and RMSE indicate a reduced voxel‐wise intensity error.

### 3.3 Dosimetric comparison

We observed an excellent dose agreement between the sCTs and reference CTs. The 3D gamma analysis, using a 2%/2 mm criterion, resulted in a median pass rate of 99.8% [99.5%–99.9%] across the cohort, with a similarly high concordance at the 1%/1 mm criterion with a median pass rate of 99.5% [98.8%–99.8%], see Table 4. The global voxel‐wise dose differences were small, where the MADD was 0.036 Gy [0.036–0.061], the 95th‐percentile absolute difference was 0.088 Gy [0.035–0.131 Gy], and the median fraction of voxels within ± 0.1 Gy was 95.9% [92.5%–99.2%]. The DVH differences for the full cohort (Table 5) were small, and all evaluated clinical dose constraints remained fulfilled after recalculation on the sCT.

**TABLE 4:** Global dose agreement metrics across all 19 patients.

Metric	Median *[range]*	Unit
Gamma 1%/1 mm	99.5 *[98.8–99.8]*	%
Gamma 2%/2 mm	99.8 *[99.5–99.9]*	%
Dice 95% dose	0.996 *[0.973–0.999]*	
Dice 50% dose	0.997 *[0.994–0.999]*	
Mean dose difference	0.025 *[0.008–0.045]*	Gy
Mean absolute dose difference	0.036 *[0.016–0.061]*	Gy
95th percentile absolute difference	0.088 *[0.035–0.131]*	Gy
Fraction within ± 0.1 Gy	95.9 *[92.5–99.2]*	%
Dose similarity index	0.990 *[0.985–0.994]*	

**TABLE 5:** Differences in DVH parameters between CT and sCT dose calculations across all 19 patients.

Volume	∆D_Max_ [Gy]	∆D_Mean_ [Gy]	∆D_98%_ [Gy]	∆D_50%_ [Gy]	∆D_2%_ [Gy]
Bladder	0.08 ± 0.14	0.01 ± 0.03	0.00 ± 0.01	0.02 ± 0.05	0.10 ± 0.14
External	−0.05 ± 0.38	0.00 ± 0.02	0.00 ± 0.00	0.00 ± 0.01	0.02 ± 0.06
FemoralHead_L	0.03 ± 0.08	0.01 ± 0.02	0.00 ± 0.01	0.01 ± 0.02	0.01 ± 0.06
FemoralHead_R	0.02 ± 0.06	0.01 ± 0.02	0.00 ± 0.01	0.01 ± 0.02	0.01 ± 0.04
PTV	−0.05 ± 0.38	0.05 ± 0.11	0.04 ± 0.11	0.05 ± 0.11	−0.06 ± 0.27
Penile bulb	0.03 ± 0.08	0.03 ± 0.05	0.01 ± 0.03	0.02 ± 0.05	0.04 ± 0.09
Prostate	0.03 ± 0.14	0.05 ± 0.11	0.07 ± 0.11	0.05 ± 0.12	0.03 ± 0.12
Rectum	−0.12 ± 0.64	0.02 ± 0.06	0.00 ± 0.00	0.05 ± 0.07	−0.10 ± 0.56
Seminal vesicles	−0.02 ± 0.24	0.00 ± 0.07	0.01 ± 0.06	0.00 ± 0.07	0.01 ± 0.12

Volume	∆D_0.5cc_ [Gy]	∆D_2cc_ [Gy]	∆V_30 Gy_ [%]	∆V_36 Gy_ [%]	∆V_40 Gy_ [%]
Bladder	0.10 ± 0.13	0.10 ± 0.15	0.05 ± 0.08	0.06 ± 0.11	0.09 ± 0.17
External	−0.04 ± 0.38	−0.01 ± 0.26	0.00 ± 0.01	0.00 ± 0.01	0.00 ± 0.01
FemoralHead_L	0.02 ± 0.06	0.01 ± 0.05	0.00 ± 0.00	0.00 ± 0.00	0.00 ± 0.00
FemoralHead_R	0.01 ± 0.05	0.01 ± 0.04	0.00 ± 0.00	0.00 ± 0.00	0.00 ± 0.00
PTV	−0.04 ± 0.36	0.00 ± 0.22	0.00 ± 0.00	0.00 ± 0.00	0.00 ± 0.01
Penile bulb	0.04 ± 0.08	0.03 ± 0.05	0.01 ± 0.02	0.01 ± 0.03	0.00 ± 0.01
Prostate	0.03 ± 0.12	0.04 ± 0.11	0.00 ± 0.00	0.00 ± 0.00	0.00 ± 0.00
Rectum	−0.09 ± 0.55	−0.13 ± 0.60	0.01 ± 0.12	−0.07 ± 0.35	0.11 ± 0.74
Seminal vesicles	0.02 ± 0.10	−0.01 ± 0.11	0.02 ± 0.18	0.04 ± 0.30	0.02 ± 0.61

*Note*: Differences in DVH parameters between CT and sCT dose calculations across all 19 patients, for STAPLE segmentations, reported as mean ± SD. The difference delta, ∆ = CT—sCT, are in units of Gy or %, where positive values indicate higher doses on CT‐based plans. The planning target volume (PTV), is the prostate with an added margin of 5 mm.

## 4 DISCUSSION

The AI‐segmentations together with the AI‐generated sCT, from the Gold Atlas dataset, resulted in a workflow that performed comparable to expert‐defined segmentations and reference CT images. The observed differences in segmentation, image and dose were minor and primarily within the expected interobserver variability range. These results further support the suitability of AI‐segmentation and MRI‐only workflows for integration into prostate and pelvic RT.

The statistically significant segmentation differences were generally small in absolute magnitude. The bladder showed significant but small shifts in several metrics, while the penile bulb showed a larger positive volume difference and lower precision, consistent with a tendency toward larger AI‐generated contours for this structure. The prostate and seminal vesicles also showed significant volume or recall‐related differences. Importantly, the leave‐one‐out kappa test indicated that the AI‐segmentations were not distinguishable from the manual observers, suggesting that these systematic shifts remained within the range of observer variability. The clinical importance of segmentation differences also depends on the role of each structure in plan optimization. Differences in target structures such as the prostate and seminal vesicles may have greater dosimetric relevance than differences in less critical OARs such as the penile bulb.

We observed excellent dose agreement between the sCTs and CTs, with a high 3D gamma pass rate and minimal voxel‐wise differences. The differences in the DVHs were small across all targets and OARs (Table 5), and all evaluated clinical dose constraints remained fulfilled after recalculation on the sCT. The variability in dose to the rectum was slightly larger than most organs, likely due to differences in rectal filling and transient air between imaging occasions. Image+ does not explicitly include transient pelvic air pockets in the generated sCT, which is consistent with clinical practice where unstable rectal air is commonly overwritten or removed for dose calculation. This may contribute to local HU differences near the rectum but did not result in clinically relevant dose deviations in this study. Since the same STAPLE‐based structure set and RayStation Generic CT calibration curve were used for both CT and sCT dose calculations, the dosimetric comparison primarily reflects differences in HU representation between the CT and sCT. However, local differences may still arise from residual registration uncertainty, differences in transient pelvic air, and the finite accuracy of sCT HU assignment.

Our results are consistent with recent studies on MRI‐only RT workflows. Reported mean absolute errors for pelvic sCT are typically in the range of 30–40 HU^3^^,^22, which aligns with our observed median MAE of 34.0 HU. Similarly, reported gamma pass rates exceed 96% in the whole pelvis^22^ and 97.9%–99.8% for the prostate^22^, ^23^ for 2%/2 mm criteria, comparable to our observed 99.8%. Reported segmentation accuracy for the prostate reaches Dice coefficients of 0.84–0.95^23^, ^24^ while more variable structures such as the rectum and seminal vesicles show lower agreement, often within interobserver variability.

A limitation of our study is that the Gold Atlas dataset is a few years old. Although it is a comprehensive and useful dataset, segmentation definitions and clinical practice may have changed slightly for less standardized organs such as the penile bulb and seminal vesicles. This is a probable contributor to the observed volume differences for the penile bulb, prostate, and seminal vesicles. A change in the seminal vesicle segmentation may also influence the prostate volume because of the proximity of the structures. However, the results from the leave‐one‐out kappa test confirmed that there was no instance where the AI‐segmentation was deemed an outlier. The relatively small cohort size (*n* = 19) and variability in imaging protocols across institutions may influence generalizability. However, this variability reflects real‐world clinical conditions and supports evaluation across different acquisition settings. Considering the well‐characterized and high quality of the Gold Atlas dataset, small differences in definition should not affect the overall findings.

## 5 CONCLUSION

The evaluated AI‐segmentations and AI‐generated sCTs (MVision) achieved high quantitative agreement with the expert‐defined manual segmentations and reference CTs from the Gold Atlas dataset. These findings support their potential integration into MRI‐only radiotherapy planning and evaluation workflows.

## AUTHOR CONTRIBUTIONS

**Joakim Jonsson**: Conceptualization; methodology; software; validation; formal analysis; investigation; resources; data curation; writing—original draft; writing—review and editing; supervision; project administration; funding acquisition. **Amanda Östensson**: Conceptualization; methodology; software; validation; formal analysis; investigation; data curation; writing—original draft; writing—review and editing; visualization.

## CONFLICT OF INTEREST STATEMENT

We would like to disclose that Joakim Jonsson is a part‐owner of Hero Imaging AB, which produces the software used for parts of the analysis, that is, not the software that has been evaluated. It has not influenced the objectivity of the analysis.

## ETHICAL APPROVAL

This study uses a publicly available dataset, which is fully anonymized. No identifiable personal information was accessed or used. According to applicable ethical guidelines and regulations, ethical approval and informed consent were not required for this study.

## Supporting information



Supporting Information

## Data Availability

The data that support the findings of this study are available from the corresponding author upon reasonable request.
