# Supplementary material for: Evaluation of AI‐based segmentation and synthetic CT generation for MRI‐only prostate radiotherapy planning
Source: J Appl Clin Med Phys. 2026 Aug 25;27(9):e70765. doi: 10.1002/acm2.70765 (PMC13504376; doi:10.1002/acm2.70765)
Supplement: Supplementary file 1 [file ACM2-27-e70765-s001.docx]

***Table S1****. Mean values for the segmentation metrics evaluated for the five organs.*

| **Metric** | **Bladder** | | **Penile Bulb** | | **Prostate** | | **Rectum** | | **Seminal Vesicles** | |
| --- | --- | --- | --- | --- | --- | --- | --- | --- | --- | --- |
|  | MVision | User | MVision | User | MVision | User | MVision | User | MVision | User |
| ASSD (mm) | 1.26 | 0.82 | 1.56 | 1.36 | 1.18 | 1.11 | 1.84 | 2.12 | 1.36 | 1.32 |
| Centroid distance (mm) | 1.61 | 0.83 | 1.98 | 2.25 | 1.67 | 1.20 | 7.84 | 8.95 | 2.55 | 2.22 |
| Dice | 0.92 | 0.95 | 0.72 | 0.76 | 0.90 | 0.90 | 0.86 | 0.85 | 0.76 | 0.78 |
| HD95 (mm) | 4.51 | 2.71 | 4.86 | 5.66 | 3.37 | 3.96 | 13.32 | 15.42 | 5.31 | 6.15 |
| Precision | 0.90 | 0.93 | 0.61 | 0.70 | 0.87 | 0.86 | 0.86 | 0.85 | 0.74 | 0.71 |
| Recall | 0.95 | 0.97 | 0.90 | 0.88 | 0.93 | 0.95 | 0.86 | 0.86 | 0.80 | 0.88 |
| Surface Dice | 0.87 | 0.91 | 0.74 | 0.77 | 0.84 | 0.85 | 0.86 | 0.84 | 0.78 | 0.81 |
| Difference in volume (%) | 0.52 | | 15.33 | | -5.19 | | -1.91 | | -13.83 | |

*Result of the segmentation performance metrics across all patients, stratified by organ. The columns show the mean value for each metric, for AI-segmentations (MVision) to the left and the five expert segmentations (User) to the right for each organ. The last column shows the volume difference between MVision and the Users in percentage.*
